# Antimicrobial properties of *Anopheles albimanus* pericardial cells

**DOI:** 10.1007/s00441-012-1505-6

**Published:** 2012-11-16

**Authors:** Salvador Hernández-Martínez, Humberto Lanz-Mendoza, Jesús Martínez-Barnetche, Mario H. Rodríguez

**Affiliations:** Centro de Investigaciones Sobre Enfermedades Infecciosas, Instituto Nacional de Salud Pública, Av. Universidad 655, Col. Sta. María Ahuacatitlan, CP 62508 Cuernavaca, Morelos México

**Keywords:** Pericardial cells, Immune response, Dorsal vessel, Mosquito, *Anopheles albimanus* (Insecta)

## Abstract

Insect pericardial cells (PCs) are strategically located along the dorsal vessel where they encounter a high hemolymph flow enabling them to undertake their osmoregulatory, detoxifying, and scavenging functions. In this location, PCs also encounter foreign molecules and microorganisms. The response of PCs of the mosquito *Anopheles albimanus*, one of the most important *Plasmodium vivax* vectors in Mexico and Latin America, to *Saccharomyces cerevisiae* was analyzed by using biochemical, cellular, ultrastructural, and bioinformatics approaches. Immune gene transcripts were identified in the PC transcriptome of *A. albimanus*. PCs responded to the presence of yeast and zymosan with increased lysosomal and phosphatase activities and produced lytic activity against bacteria. Our results indicate that mosquito PCs play a key role in the neutralization and elimination of pathogens.

## Introduction

Insects, including mosquitoes, display an efficient innate immunity aimed at the isolation and destruction of invaders. Their innate immune responses are overall similar to that of vertebrates (Schmid-Hempel 2005) and comprise functional processes for pathogen recognition, signaling and transduction pathways, and effector mechanism processes (Baton et al. 2008). Pathogen-associated molecular patterns (PAMPs) are detected by patterns recognition receptors (PRRs; Akira et al. 2001; Medzhitov and Janeway 2002; Dimopoulos et al. 2002) that often trigger a signal amplification system through the activation of serine protease cascades modulated by serine protease inhibitors (Waterhouse et al. 2007).

A variety of recognition and signal pathways determine specialized responses (Schulenburg et al. 2007) mediated by at least three transduction pathways. Toll components are activated by Gram-positive bacteria, fungi (Christophides et al. 2004), and virus (Sanders et al. 2005; Ramirez and Dimopoulos 2010). The immune deficiency (Imd) pathway responds to Gram-positive bacteria (Meister et al. 2005), and both Toll and Imd and the Janus-kinase-signal transducer and activator of transcription pathway (JAK-STAT) participate in responses against Plasmodia (Cirimotich et al. 2010; Garver et al. 2009; Gupta et al. 2009; Bahia et al. 2011).

The outcome is the activation of a variety of effector molecules and processes. Toll and Imd activation leads to the transcriptional induction of several antimicrobial peptides (AMP; Cirimotich et al. 2010; Lemaitre et al. 1995, 1996; Lowenberger et al. 1995; Michel et al. 2001; Richman et al. 1997; Vizioli et al. 2001; Waterhouse et al. 2007), which are mainly produced in the fat body and released into the hemolymph (Tzou et al. 2000). Cellular defenses are mediated by hemocytes and include phagocytosis, nodulation, and encapsulation (Hernández-Martínez et al. 2002; Hillyer and Christensen 2002; Hillyer et al. 2003; Lavine and Strand 2002; Schmidt et al. 2001). Hemocytes also produce humoral molecules, and other organs are involved in cellular and humoral responses, as exemplified by AMP and other responses in the midgut and salivary glands (Dimopoulos et al. 2000).

Hemolymph, which contains nutrients, waste, signal molecules, and immune factors is distributed to all insect body structures in an open circulatory system. Although secondary pulsatile organs are located in other parts of the body, hemolymph is mainly pumped by the dorsal vessel in alternating anterior (toward the head) and posterior directions. This vessel is a tubular organ located medially on the dorsal wall of the tegument of the insect and extends from the abdominal end to the head (Martins et al. 2011). The abdominal portion (heart) is pulsatile and presents wall openings (ostia) that function as valves during hemolymph circulation. The aorta, which lies in the thorax, has no ostia and simply conducts the hemolymph to the anterior part of the body. The heart is tethered to the abdominal posterior wall by six pairs of alary muscles. These muscles also maintain the shape of the heart and probably expand the vessel during diastole (Glenn et al. 2010).

Pericardial cells (PCs), also named nephrocytes (Andereck et al. 2010; Chapman 1998), are present in single lines along the dorsal vessel. They have peripheral finger-like projections bordering a labyrinthine channel system and surface invaginations (Martins et al. 2011; Jones 1977; Rizki 1978). Molecules are taken from the hemolymph by pinocytosis and, after degradation, might be returned to the hemolymph by exocytosis (Andereck et al. 2010; Chapman 1998; Glenn et al. 2010; Martoja and Ballan-Dufrancais 1984).

Having osmoregulatory and detoxifying functions, PCs are strategically positioned in a place of high hemolymph flow, where they are also highly exposed to foreign molecules and microorganisms. Accordingly, immune response markers identified in mosquito PCs suggest that these cells can also participate in the neutralization of pathogens. Serpins, a family of proteins that participate in insect immune regulation, are expressed in the nuclei and cytoplasm of PCs of the *Anopheles* mosquito after a bacterial infection (Danielli et al. 2003). A member of the STAT (signal transducers and activators of transcription) family is constitutively present in the cytoplasm and nuclei of PCs (Barillas-Mury et al. 1999). *Sp22D*, a modular serine protease has been identified in the cytoplasm of PCs both in naive and bacteria-challenged mosquitoes. In addition, defensin accumulates in these cells several hours after bacterial challenge (Danielli et al. 2000). Similar observations have been made with the related thioester-containing protein (TEP-I; Levashina et al. 2001). However, no data are available as to whether these proteins originate from endogenous production or are taken from the hemolymph.

We provide herein histological, histochemical, ultrastructural, and bioinformatic evidence that indicates the participation of *Anopheles albimanus* PCs during the elimination of pathogens.

## Materials and methods

### Mosquitoes and microorganisms

White-striped pupal-phenotype 2 to 3-day-old adult female *A. albimanus* mosquitoes, a highly susceptible strain to *Plasmodium vivax* infection (Chan et al. 1994), from the insectary of the Instituto Nacional de Salud Pública (INSP) in Cuernavaca, Morelos, Mexico, were used. The mosquitoes were maintained as previously described (Chan et al. 1994). Briefly, adult mosquito rearing and maintenance were carried out under insectary conditions at 28°C, 80% relative humidity, with a 12-h light/12-h dark photoperiod. Mosquitoes were fed with cotton pads soaked with 3% sucrose solution ad libitum.

*Saccharomyces cerevisiae* and *Microccocus lysodeikticus* (both from Sigma, St. Louis, Mo., USA) were used. To prevent yeast proliferation, they were killed by heat in a boiling bath for 3 min, before inoculation into the mosquitoes.

### Mosquito inoculations and heart dissections

Inoculation needles were made from 100-μl micro-capillary tubes drawn into fine tips by hand and attached to pipette pumps. Mosquitoes were briefly cold-anesthetized on ice and injected with 0.25 μl RPMI 1640 culture medium (with added phenol red; Gibco BRL, Grand Island, N.Y., USA) containing approximately 2×10^3^ yeast or with the same volume of the soluble fraction (β-1,3 glucan) of zymosan (Sigma) at 100 μg glucose equivalents/ml (Lanz et al. 1993). Control mosquitoes were injected with 0.25 μl RPMI alone or non-injected. Inoculations were carried out through the pleural membrane, between the fourth and fifth abdominal segments. Mosquitoes were maintained at room temperature (RT) for 2 h.

No fat body cells were observed to be part of the heart (abdominal section of dorsal vessel). After disruption of the alary muscles, the heart with attached PCs was removed from the abdomen in a drop of potassium-phosphate-buffered saline (PBS, 140 mM NaCl, 2.6 mM KCl, 1.5 mM KH_2_PO_4_, 20.4 mM Na_2_HPO_4_, pH 7.2). Some fat body cells contaminated the heart preparations during their extraction, but the preparations were carefully washed in a drop of PBS under a dissecting microscope until visually no contaminating material was evident. These preparations (now referred as clean hearts) consisted of only the dorsal vessel and the attached PCs.

### Histochemical and enzymatic assays

Clean hearts from RPMI-, yeast-, and zymosan-injected mosquitoes were used to investigate lysosomal, acid phosphatase, and lytic activities. They were obtained as above and incubated for 10 min at RT in 0.01% neutral red (Sigma) in PBS, pH 3.5, washed in PBS, pH 7.2, and observed directly via an E600 Nikon bright-field microscope. Red staining of the cytoplasm evidenced the presence of lysosomal activity (Hernández-Martínez et al. 2002; Luckhart et al. 1992). Relative lysosomal activity was assessed in groups of ten clean hearts in five independent experiments. After incubation in neutral red, clean hearts were lysed by three cycles of freeze/thaw (−96/+37 °C) in PBS, pH 3.5 and centrifuged for 10 min at 10,000*g*. The absorbance of the supernatants was read at 540 nm by using an ELISA reader (Labsystems Multiskan, Vienna, Va., USA). Additional experiments were conducted with fixed clean hearts to eliminate the possibility that the neutral red label observed in live hearts in vitro resulted from nonspecific uptake from the incubation solution. Clean hearts of RPMI- and zymosan-injected-mosquitoes in a drop of PBS, pH 7.2, on slides were placed in a chamber and fixed with paraformaldehyde (37% solution) vapors for 30 min (Hernández-Martínez et al. 2002). These samples were incubated with neutral red as described above.

To investigate acid phosphatase activity, histochemical studies were conducted in isolated clean hearts of mosquitoes treated with yeast or zymosan. Two hours after treatment, clean hearts were obtained and fixed for 30 s in a mixture of citrate/acetone/formaldehyde (5:3:2), washed with de-ionized water, and incubated for 1 h at 37 °C in phosphate AS-BI naphthol (Sigma) with or without sodium tartrate (as a control). Samples were analyzed by bright-field microscopy. The presence of acid phosphatase activity was identified by the presence of brown-stained granules in the cytoplasm (as indicated in data sheet of the reagent; Sigma).

Relative acid phosphatase activity was investigated in groups of five clean hearts in five independent experiments by using a SPINREACT KIT 1001121 (Girona, Spain). Clean hearts from yeast- or zymosan-injected mosquito and controls (RPMI-injected or non-treated) were incubated for 10 min in 200 μl phosphate AS-BI naphthol (with or without sodium tartrate) at RT in a 96-well ELISA plate. After incubation, hearts were removed, and the absorbance in the solution was read at 405 nm by using an ELISA reader (Labsystems Multiskan). Differences among mean absorbance values were evaluated by using an analysis of variance (Zar 1999).

Additional experiments were conducted to investigate PC phosphatase activity in clean hearts stimulated in vitro. Groups of five clean hearts, obtained in RPMI culture medium, were incubated in 200 μl RPMI containing zymosan at 100 μg glucose equivalent/ml (Lanz et al. 1993) or in 200 μl RPMI (control). After 2 h, the medium was removed, and samples were incubated with phosphatase acid substrate, as described before. In this case, the absorbance was recorded every 10 min over 2 h.

PC lytic activity was measured by adapting an *M. luteus* lytic assay previously described by Shugar (1952). Groups of ten mosquitoes were injected with either 0.25 μl RPMI containing zymosan or 0.25 μl RPMI or were non-treated. After 2 h, clean hearts were obtained (as described above) and lysed in 55 μl PBS by three rapid freeze/thaw (−96/+37 °C) cycles and centrifuged at 14,000*g*. The supernatant was recovered; 50 μl of this supernatant was incubated with 50 μl live *M. lysodeikticus* in PBS (360 μg/ml). The absorbance at 450 nm was recorded every 2 min, over 40 min. The lysis of bacteria resulted in a decrease of the absorbance.

An additional experiment was performed to investigate whether possible contaminating fat body cells or the vessel tissues were responsible for the lytic activity observed in clean heart preparations. Groups of 10 mosquitoes were injected with zymosan as described above, and after 2 h, their hearts were obtained. In the first group, clean hearts were recovered in 55 μl PBS. In the second group, contaminating tissues (mainly fat body) obtained during the heart cleaning process were recovered in the same volume of PBS. In the third group, PCs were removed/destroyed to obtain dorsal vessels (PCs-free), which were recovered in the same volume of PBS. No PCs alone were collected, most of them being destroyed during removal. All samples were lysed and included in lytic activity assays as described before.

### Transmission electron microscopy

Clean hearts from yeast-injected mosquitoes were obtained as described before and immediately fixed in 4% paraformaldehyde/0.1% glutaraldehyde in PBS. After 24 h at RT, the specimens were washed with PBS, dehydrated by using progressive ethanol concentrations, and embedded in Epon 812 resin (Sigma). Semi-thin sections (0.5 μm) were stained with toluidine blue. Thin sections were obtained by using an RMC ultramicrotome (Model MTX, Tucson, Ariz., USA) in order to observe PCs under light microscopy. At least two areas of each mosquito (10 mosquitoes) were selected in semi-thin sections for preparation for electron microscopy. Thin sections (80 nm) were contrasted with 5% uranyl acetate and lead citrate (80 mM lead nitrate, 120 mM sodium citrate, and 160 mM NaOH; Reynolds 1963) and examined in a Jeol transmission electron microscope (JEM-1011; Hernández et al. 1999). The presence of yeast interacting with PC was investigated.

### Transcriptome analysis

We previously reported a transcriptomic analysis of adult *A. albimanus* female mosquitoes with conventional and Next Generation Sequencing of cDNA libraries derived from various mosquito tissues, including the dorsal vessel (Martinez-Barnetche et al. 2012). To search for immunity-related transcripts expressed in the dorsal vessel libraries, the reported 16,699-annotated transcript assembly dataset was used as a reference transcriptome to map 454 reads from zymosan- and PBS-inoculated mosquitoes (Short Read Archive Accession: SRX144325) with GS Reference Mapper 2.6 software (454 Life Sciences) in the cDNA-mapping mode. Reference transcripts mapped by at least two sets of dorsal-vessel-derived 454 reads covering at least 10% of their length were considered as being expressed in the dorsal vessel. Among these transcripts, we investigated which ones were included in a data set of 82 immunity-related genes previously identified in the whole *A. albimanus* dataset (Martinez-Barnetche et al. 2012).

## Results

### PCs response to *Saccharomyces cerevisiae*

**Fig. 1:**
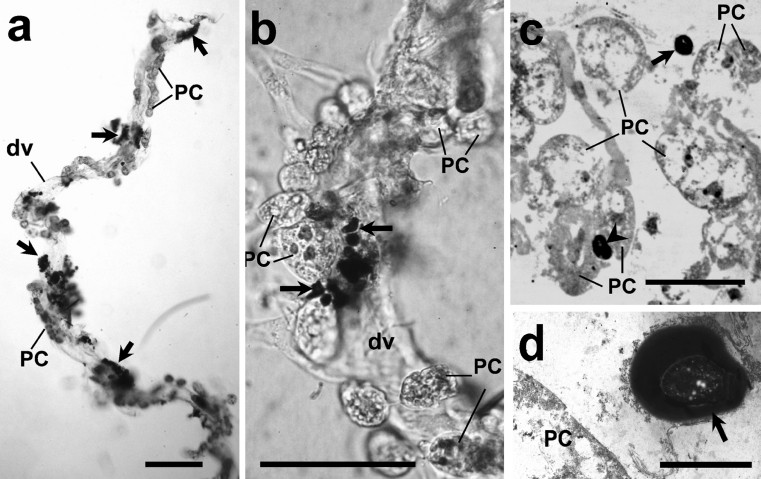
Clean hearts removed from female *Anopheles albimanus* mosquitoes injected with *Saccharomyces cerevisiae* and observed by light microscopy in fresh samples (**a**, **b**), in semi-thin sections stained with toluidine blue (**c**), and by transmission electron microscopy (**d**). Most of the yeast were aggregated and melanized (*arrows*) around the mosquito heart, but none were seen inside pericardial cells (*PC*). **a** Low magnification of a whole isolated heart showing pericardial cells (*PC*) and aggregated yeast (*arrows*) around dorsal vessel (*dv*). **b** Details of aggregated yeast (*arrows*) shown at higher magnification. **c** In semi-thin sections, yeast were seen close to PC (*arrow*) but were not phagocytozed (*arrowhead* one yeast cell lying between two PC). **d** Details of melanized yeast close to PC. *Bars* 200 μm (**a, b**), 50 μm (**c**), 5 μm (**d**)

Aggregated *S. cerevisiae* were observed in the hemocoel of *A. albimanus* as early as 10 min after inoculation (data not shown). However, in 20 examined mosquitoes, melanized yeast aggregation was more evident by 1 h post-injection. Most of them were localized around dorsal vessels (Fig. 1a-c). Transmission electron microscopy revealed no yeast particles inside PCs or the dorsal vessel lumen (Fig. 1d).

### Lysosomal, acid phosphatase, and lytic activities in PC after challenge

**Fig. 2:**
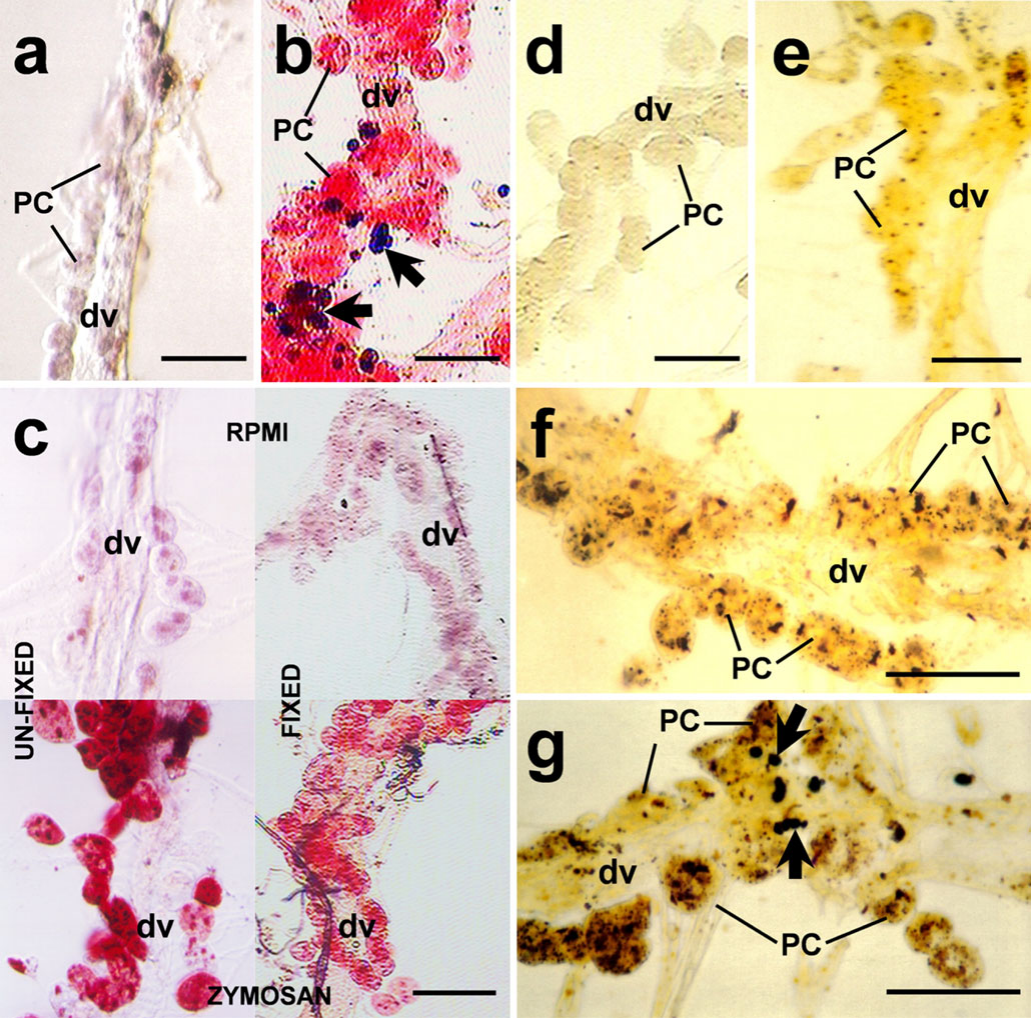
Lysosomal (**a-c**) and acid phosphatase (**d-g**) activities induced in *A. albimanus* PCs from isolated clean hearts after a *S. cerevisiae* or zymosan challenge. **a** Clean heart from non-treated mosquitoes showing the vessel (*dv*) with no staining of neutral red in pericardial cells (*PC*). **b** Clean heart from yeast-inoculated showing strong staining in PCs and melanized yeast aggregations (*arrows*). **c** PCs stained with neutral red in zymosan-injected mosquitoes (*bottom left*, *bottom right*) indicating strong lysosomal activity in these cells compared with control RPMI-injected (*top left*, *top right*). Staining was performed in isolated unfixed (*left top*, *left bottom*) and fixed (*right top*, *right bottom*) clean hearts to eliminate the possibility of unspecific neutral red uptake by PCs. **d** Clean hearts from zymosan-injected mosquitoes, treated with sodium tartrate before the addition of the substrate, were negative. **e** Samples from RPMI-injected mosquitoes showed sparse labeling. **f**, **g** Acid phosphatase activity induced in PCs from zymosan- or yeast-injected mosquitoes, respectively. Melanized and aggregated yeast are indicated by *arrows* in **g**. *Bars* 100 μm

**Fig. 3:**
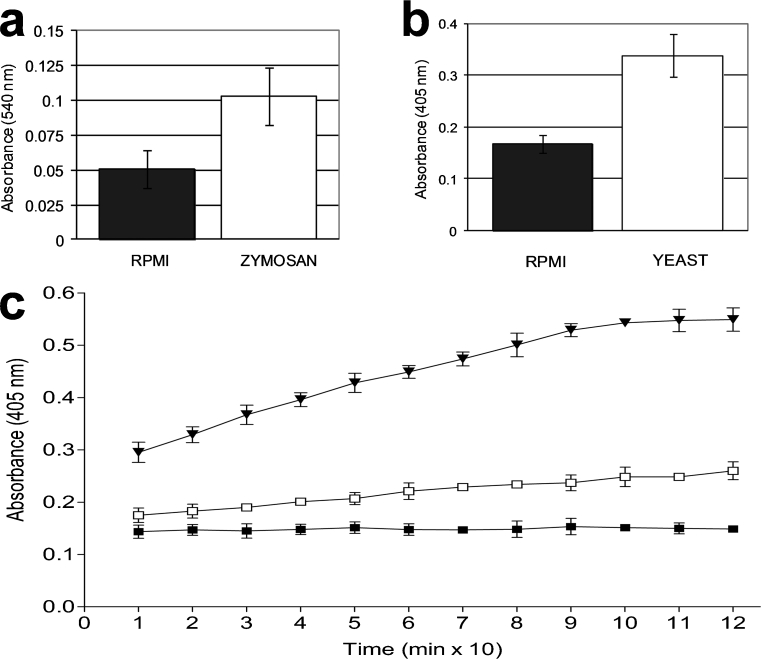
Relative enzyme activities, namely lysosomal (**a**) and acid phosphatase (**b**), confirmed the histochemical observations. Both enzyme activities were twice as high in clean hearts of challenged mosquitoes (*P*=0.001) than in controls. Relative phosphatase acid activity (**c**) in clean hearts challenged in vitro. Clean hearts were first incubated for 2 h in RPMI medium containing zymosan and then incubated with phosphatase acid substrate for two additional hours recording the absorbance every 10 min. In vitro stimulus with zymosan induced higher enzyme activity than in the RPMI control. Each value represents the mean ± SD absorbance of three independent assays with five clean hearts in each group (*black triangle* zymosan, *white square* RPMI, *black square* preparations without hearts)

PCs responded strongly to yeasts or zymosan. In histochemical unfixed preparations, strong staining was observed with neutral red in PCs of mosquitoes challenged with yeasts or zymosan, compared with those untreated and with RPMI-treated controls (Fig. 2a, b, c left). However, not all PCs were stained with neutral red, indicating that some cells were not exposed to the stimuli or the existence of a variety of cell subpopulations. Assays performed in fixed clean hearts (after the challenge) showed similar results (Fig. 2c right), confirming that lysosomal activity was induced within PCs. The relative lysosomal activity, as measured by the supernatant absorbance, was higher (50%) in yeast-injected (data not shown) and zymosan-injected mosquitoes than that in the control group (0.103±0.021 and 0.051±0.014, respectively; *P*=0.0001, five independent experiments, Fig. 3a).

In histochemical preparations of clean heart treated with phosphate AS-BI naphthol as a substrate to investigate acid phosphatase activity, PCs from RPMI-injected mosquitoes presented sparse labeling (Fig. 2e), but samples from zymosan- and yeast-injected (Fig. 2f, g) mosquitoes presented intense labeling. Clean hearts from mosquitoes injected with zymosan but treated with sodium tartrate before incubation with AS-BI naphthol showed no staining (Fig. 2d). The relative phosphatase activity was similar in zymosan-injected (data not shown) and yeast-injected mosquitoes, but higher than that in control samples injected with RPMI (0.338±0.04 vs. 0.168±0.017; *P*=0.0001, five independent experiments, Fig. 3b). Assays performed in vitro showed similar results. In-vitro-stimulated clean hearts showed higher acid phosphatase activity than the controls, indicating that this activity did not represent enzyme uptake from the hemolymph by PCs (Fig. 3c).

**Fig. 4:**
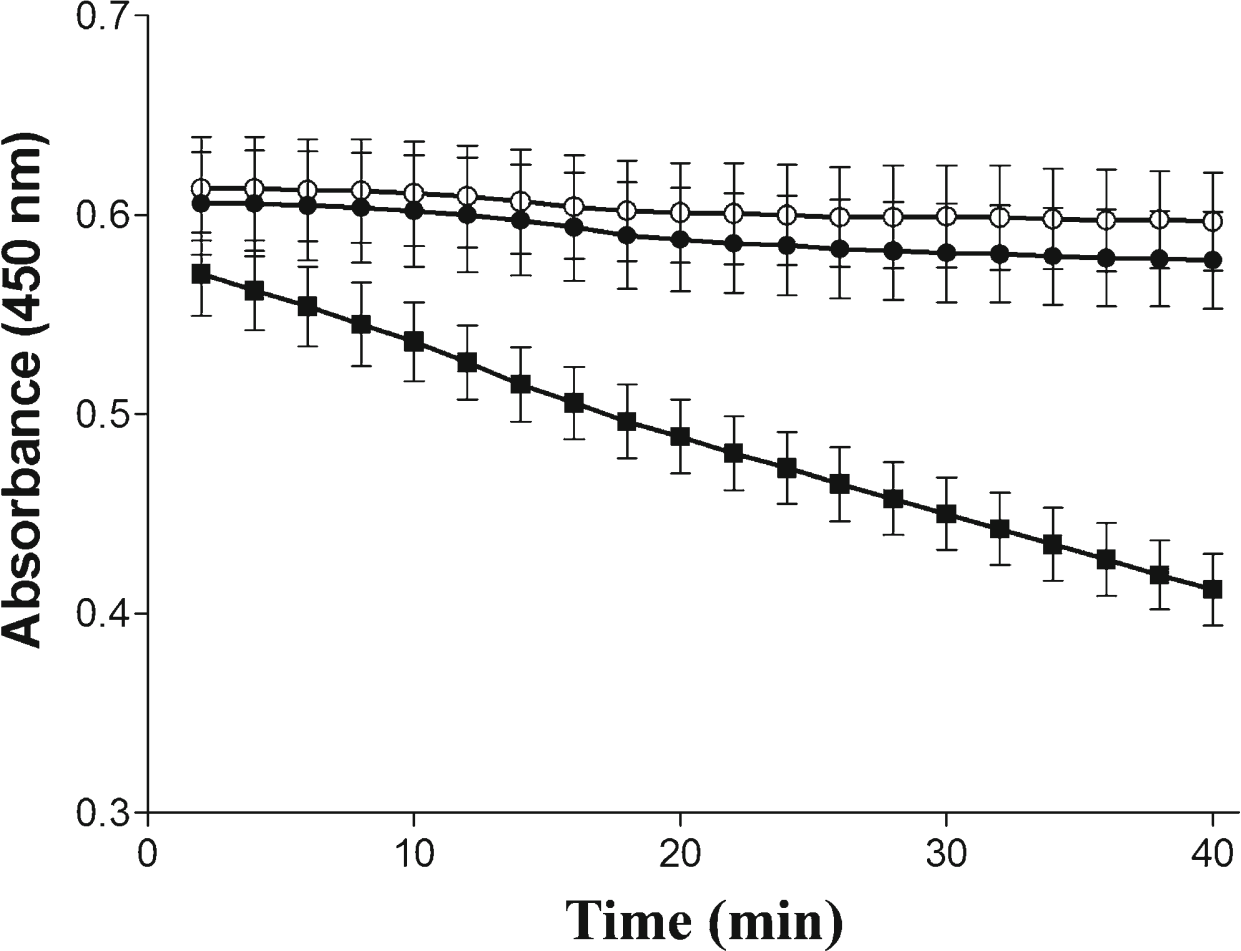
Lytic activity in clean hearts of *A. albimanus* challenged with zymosan. Mosquitoes were injected with either 1 μl medium (RPMI) containing zymosan (100 μg glucose equivalents/ml; *black squares*) or 1 μl RPMI (*black circles*); non-injected control (*white circles*). After 2 h, clean hearts were obtained and lysed in potassium phosphate buffer. Clean heart extract (50 μl) from each group was incubated with 50 μl *Microccocus lysodeikticus* (360 μg/ml). The absorbance at 450 nm was recorded every 2 min. Each value represents the mean ± SD absorbance of three independent assays with ten clean hearts in each group. Decrease of absorbance indicates the amount of bacteria lysed

Lytic activity against *M. lysodeikticus* was documented (progressive decrease in the absorbance of PCs preparations) only in clean heart extracts from zymosan-treated mosquitoes (Fig. 4). The absorbance of clean heart extracts from non- or RPMI-treated mosquitoes incubated with bacteria (0.597±0.023, 0.577±0.24, respectively), did not decrease after 40 min of incubation compared with zymosan-treated samples (412±0.018, *P*=0.0001, *n*=3).

**Fig. 5:**
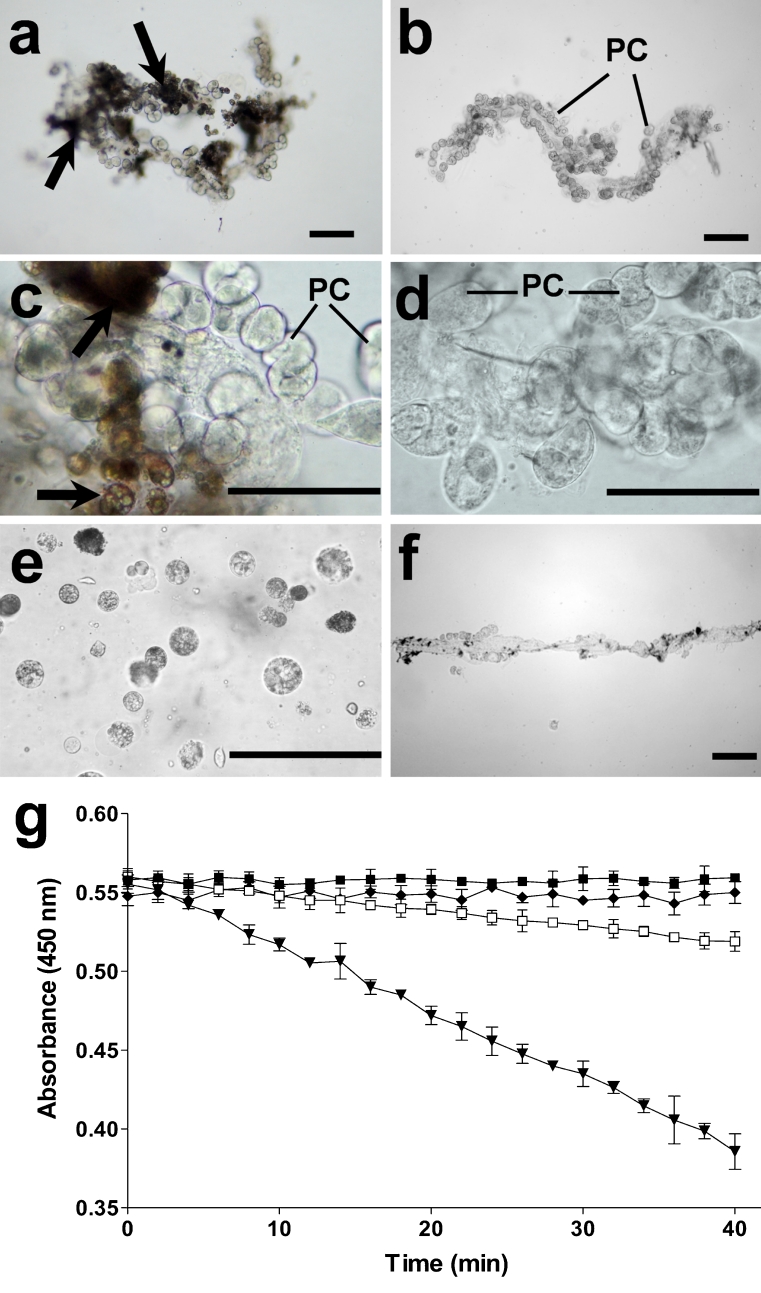
Heart preparations from *A. albimanus* as used in lytic assays. **a**, **c** Newly obtained hearts contained fat body cells (*arrows*). **b**, **d** After being carefully washed with PBS, hearts were free of fat body cells. **e** Removed fat body cells from hearts were collected for assays. **f** Dorsal vessels free of PCs and clean hearts (see **b**) were collected for the lytic assay. **g** Lytic activity against *M. lysodeikticus* is produced only by clean hearts. The absorbance at 450 nm was recorded every 2 min. Each value represents the mean ± SD absorbance of three independent assays with ten samples in each group (*white squares* fat body, *black diamonds* vessels free of PCs, *black downward-pointing triangles* clean hearts, *black squares* sample of *M. lysodeikticus* alone). *Bars* 100 μm

The tissues used in experiments to compare the lytic activity of cell types in heart preparations are depicted in Fig. 5: hearts extracted from the insect body were initially contaminated with fat body cells (seen as brownish cells in Fig. 5a, c). The preparations were carefully washed to remove fat body cells, and clean heart preparations were obtained (Fig. 5b, d). Fat body cells removed from newly obtained hearts are shown in Fig. 5e. Clean vessels were obtained after removing PCs (Fig. 5f). Lytic activity was detected only in samples of clean heart extracts (Fig. 5g). Fat body cells (removed from heart preparations) or clean vessel extracts (without PCs) did not lyse bacteria.

### Immunity-related transcripts expressed in dorsal vessel

Mapping 454 reads from the dorsal vessel into the *A. albimanus* reference transcriptome led to the identification of 3,212 reference transcripts as expressed in the dorsal vessel. Of these, 26 reference transcripts were immunity-related orthologs, as previously described (Supplemantary table, Martinez-Barnetche et al. 2012). Table 1 lists eight of these orthologs, all of which might be particularly relevant to the present results. These include transcripts coding for proteins with antimicrobial activity, such as three antimicrobial peptides (two cecropins and one defensin) and two lysozymes. In addition, two serpins and members of the Toll and Imd signaling pathway were identified (Table 1).

**Table 1:** Immunity-related transcripts expressed in *A. albimanus* heart

*A. albimanus* ID^a^	ImmunoDB ID^b^	Interpro annotation	*A. gambiae* ortholog	Gene name	Family
isotig00163_Length_628	AMP6	IPR000875: Cecropin	AGAP000694	CEC3	Cecropin
isotig00299_Length_500	AMP4	IPR000875: Cecropin	AGAP000693	CEC1	Cecropin
Locus_19472_Length_607	LYS7	IPR001916: Glycoside hydrolase	AGAP007386	LYSC7	LYSC
Locus_7217_Length_513	LYS1	IPR001916: Glycoside hydrolase	AGAP007347	LYSC1	LYSC
Locus_8032_Length_1343	TOLLPATH2	IPR015787: Interleukin-1 receptor-associated kinase 4.	AGAP002966	PLL1	PELLE SRPN-
isotig00138_Length_1045	SRPN11	IPR000215: serpin	AGAP005246	SRPN10	INNHIB SRPN-
Locus_10159_Length_1306	SRPN10	IPR000215: serpin	AGAP003139	SRPN9	INHIB
Locus_15867_Length_932	IMDPATH5	IPR000488: Death	AGAP000388	TRAF6	TRAF6

^a^Martinez-Barnetche et al. (2012)

^b^ http://cegg.unige.ch/Insecta/immunodb

## Discussion

Our histochemical observations provide support for antimicrobial defense functions for PC, additional to that of clearance and detoxification of the mosquito hemolymph (Chapman 1998; Jones 1977; Rizki 1978). These results indicate that PC respond and are activated by the presence of yeasts or a component of their cell wall, and that, as a result of their activation, PCs exhibit bacteriolytic activity. The possibility of an increased capacity of PCs to detoxify hemolymph during infections has not been investigated in this work. An increased clearance capacity of these cells would certainly confound the results attributed to immune activation; however, this possibility has been eliminated in experiments with fixed cells and in vitro experiments without hemolymph.

We present here, for the first time, evidence for the activation of PC by yeasts or zymosan. The induction of lysosomal activity in PC of yeast- or zymosan-treated mosquitoes, compared with untreated and RPMI-treated controls, was observed in situ. The possibility that increased endocytosis of the reagents by activated cells could explain our findings was eliminated by similar results in assays of fixed cells. Additionally, increased acid phosphatase activity was quantified in the supernatants of short-term cultures after the removal of in-vivo-challenged hearts. Acid phosphatase activity induced *de novo* in PCs was confirmed in assays in which clean hearts were challenged in vitro. These data suggest that this enzyme might be present as a zymogen in PCs.

We also documented that activated PCs lysed *M. lysodeikticus* (as shown in Figs. 4, 5). Using heart preparations that were carefully cleaned, we minimized the possibility that fat body cells, which are known to produce antimicrobial peptides and lysozyme (Dunn et al. 1985; Faye and Wyatt 1980; Kanost et al. 1988), were responsible for the observed bacterial lysis. Furthermore, in assays with the removed fat cells and the dorsal vessel devoid of PCs, no lytic activity was documented. At present, we have no evidence for the molecular origin of the lytic activity observed, but the production of lysozyme has been documented in PCs of *Calliphora erythrocephala* (Croosley 1972).

On the other hand, our results are indicative of a lack of phagocytic activity in these cells. Previous studies reporting phagocytosis by PC are inconclusive. Jones (1954) reported the accumulation of ammonia carmine dye into the PCs of *A. quadrimaculatus* larvae, but this was must probably the result of its uptake by pinocytosis, similar to that in vertebrate nephrons (Lameire et al. 1977). Low magnification observations of latex particles, *Escherichia coli*, and *Plasmodium berghei* sporozoites localized in the PC area of *A. gambiae* have also been interpreted as phagocytosis, but without histological documentation (Hillyer et al. 2007). However, using transmission electron microscopy, we have never observed phagocytozed yeast in *A. albimanus* PCs. Instead, most of melanized yeast were aggregated (Fig. 1, arrows) around the mosquito heart. This is compatible with the histological structure of this tissue, which is covered by a basal lamina that limits the transit of particulate material. The presence of melanized yeast near PCs does not indicate that PCs are responsible for this phenomenon, and although we cannot eliminate the possibility of the damage of these yeasts by PCs, melanization was most probably produced by hemolymph components.

Insects respond to a wide variety of foreign materials by mounting cellular and humoral processes aimed at the isolation and destruction of invaders (Beerntsen et al. 1994; Cupp et al. 1997; Gorman and Paskewitz 1997; Hoffman et al. 1999; Lavine and Strand 2002; Paskewitz and Riehle 1994). Insects lack filtration organs (e.g., liver, spleen, lymph nodes) that, in vertebrates, remove and are the main sites of immune detection of circulating invading pathogens. Traditionally, hemocytes and the fat body have been considered the main tissues responsible of the effector components of the immune response in insects (Hernández-Martínez et al. 2002; Hillyer and Christensen 2002; Hillyer et al. 2003; Lavine and Strand 2002; Schmidt et al. 2001; Tzou et al. 2000). However, an initial encounter with pathogens, through tegument wounds or within the digestive tract, might trigger locally mounted constitutive innate immune responses, such as the production of oxygen radicals and melanization (Lanz et al. 1993), and antibacterial and anti-parasite responses have been documented in the midgut and salivary glands of mosquitoes (Dimopoulos et al. 2000). PCs are strategically located on the dorsal vessel, which is exposed to a high flow of hemolymph, and can therefore encounter circulating microbial invaders and respond with defense mechanisms. For instance, only 10%–25% of the thousands of malaria sporozoites produced in oocysts invade the salivary glands, and despite attempts to elucidate the fate of the rest of the parasites (Hillyer et al. 2007), the participating mechanisms remain elusive. During their journey to the salivary glands, sporozoites are directly exposed to a repertoire of PRRs, antimicrobial peptides (Boman and Hultmark 1987; Hoffmann and Hoffmann 1990; Lowenberger et al. 1996), and other reactive molecules present in the hemolymph (Lanz-Mendoza et al. 2002). However, the extent of the participation of these immune mechanisms to their destruction awaits clarification. Cell-mediated immune responses (Bartholomay et al. 2004; Hernández-Martínez et al. 2002; Paskewitz and Shi 2005) have been proposed, and the phagocytosis of sporozoites by hemocytes has been documented (Hillyer et al. 2003). Nevertheless, the proportion of hemocytes engaged in this process is too small to account for the necessary numbers to eliminate the enormous amount of parasites that do not reach the salivary glands (Hillyer et al. 2007). Preliminary results indicate damage to sporozoites lying near to PCs (data not shown), but the participation of PCs in the process of parasite clearing awaits demonstration.

Previous studies that have documented the presence, in mosquito PCs, of molecules (serpin, defensin, STAT, and *Sp22D*) that participate in insect immune responses (Barillas-Mury et al. 1999; Danielli et al. 2003; Levashina et al. 2001) have failed to establish the site of their production, and the possibility of their uptake from the hemolymph cannot be disregarded. The transcription and presence of SPRN10 (an important regulator of the immune response) in PCs of *A. stephensi* (Lycett et al. 2004) and the transcripts coding for immunity-related effectors (AMP, lysozymes, and canonical immune signaling pathways) identified in the *A. albimanus* heart transciptome support our histochemical, biochemical, cellular, and ultrastructural observations that PCs are actively involved in the mosquito immune response, and not only in scavenging processes. However, we cannot eliminate the possibility that the immune-related transcripts identified in heart preparations are produced by other cells (dorsal vessel muscle and endothelial cells) present in these preparations. Additional studies are necessary to reject these possibilities.

In summary, we present evidence that *A. albimanus* PCs respond to the presence of invaders by mounting lytic and toxic responses. We are currently investigating the heart transcriptome to identify genes and effector molecules during the PC response against pathogens. These could provide new insights into the immune mechanisms of malaria vectors.
